# Mobile electronic versus paper case report forms in clinical trials: a randomized controlled trial

**DOI:** 10.1186/s12874-017-0429-y

**Published:** 2017-12-01

**Authors:** Robert Fleischmann, Anne-Marie Decker, Antje Kraft, Knut Mai, Sein Schmidt

**Affiliations:** 1 Clinical Research Unit, Charité Campus Mitte, Berlin Institute of Health (BIH), Charitéplatz 1, 10117 Berlin, Germany; 2 Department of Neurology, University Medicine Greifswald, Ferdinand-Sauerbruch-Straße, 17475 Greifswald, Germany

**Keywords:** REDCap, Electronic case report form, Time efficiency, Data handling

## Abstract

**Background:**

Regulations, study design complexity and amounts of collected and shared data in clinical trials render efficient data handling procedures inevitable. Recent research suggests that electronic data capture can be key in this context but evidence is insufficient. This randomized controlled parallel group study tested the hypothesis that time efficiency is superior when electronic (eCRF) instead of paper case report forms (pCRF) are used for data collection. We additionally investigated predictors of time saving effects and data integrity.

**Methods:**

This study was conducted on top of a clinical weight loss trial performed at a clinical research facility over six months. All study nurses and patients participating in the clinical trial were eligible to participate and randomly allocated to enter cross-sectional data obtained during routine visits either through pCRF or eCRF. A balanced randomization list was generated before enrolment commenced. 90 and 30 records were gathered for the time that 27 patients and 2 study nurses required to report 2025 and 2037 field values, respectively. The primary hypothesis, that eCRF use is faster than pCRF use, was tested by a two-tailed t-test. Analysis of variance and covariance were used to evaluate predictors of entry performance. Data integrity was evaluated by descriptive statistics.

**Results:**

All randomized patients were included in the study (eCRF group *n* = 13, pCRF group *n* = 14). eCRF, as compared to pCRF, data collection was associated with significant time savings  across all conditions (8.29 ± 5.15 min vs. 10.54 ± 6.98 min, *p* = .047). This effect was not defined by participant type, i.e. patients or study nurses (F_(1,112)_ = .15, *p* = .699), CRF length (F_(2,112)_ = .49, *p* = .609) or patient age (Beta = .09, *p* = .534). Additional 5.16 ± 2.83 min per CRF were saved with eCRFs due to data transcription redundancy when patients answered questionnaires directly in eCRFs. Data integrity was superior in the eCRF condition (0 versus 3 data entry errors).

**Conclusions:**

This is the first study to prove in direct comparison that using eCRFs instead of pCRFs increases time efficiency of data collection in clinical trials, irrespective of item quantity or patient age, and improves data quality.

**Trial registration:**

Clinical Trials NCT02649907.

## Background

Over the last decades, the number of clinical trials (CTs) conducted increased substantially [1]. This development was paralleled by more demanding trial regulations including data monitoring, increasing complexity of study designs and lengthy data collection protocols [2]. A similar trend towards larger amounts of more complex data to be handled in shorter time developed in clinical routine and led to the increasing use of electronic health records (EHR) [3]. A large body of evidence suggests that EHR yield both process and structural benefits [4]. The addition of mobile technology to EHR was shown to further improve data handling and increase time efficiency [5]. Mobile technology is also well-accepted and preferred over classic data handling methods by users in clinical settings [6]. Recent research suggests that improvements in EHR data handling technology can be key to meet current challenges in data handling efficiency in CTs and should thus be leveraged in electronic data capture (EDC) [7, 8].

Various studies have addressed important concerns that might be associated with the replacement of paper case report forms and questionnaires (pCRF) by their electronic counterparts (eCRF). This led to high quality evidence that using EDC in CTs is a viable method in multiple aspects. It can be used in different settings including family practices, hospitals, research facilities, field studies and participants’ homes [9–12]. pCRF and eCRF were also repeatedly shown to provide excellent internal consistency and construct equivalence, i.e. constructs can be measured equally across methods and entered values have equivalent meanings [13–15]. eCRF are furthermore preferred over paper bound methods by participants and study personnel [16, 17] and help improve data quality, particularly through a reduction of data omission errors [15, 17, 18]. Multicenter studies also benefit from easy data sharing between study sites, which allows for syntactic and semantic interoperability [19, 20]. Cost efficiency of eCRF use was evaluated in simulation studies and in the context of observational and interventional clinical trials and reported to be increased due to elimination of pCRF logistics (printing, delivery etc.), facilitation of data monitoring and time savings for study personnel [18, 21–24]. None of the studies, however, took precise time records rendering causes of time savings unclear, importantly if and which part of the data collection procedure particularly benefitted from eCRF use. It is generally assumed that data transcription redundancy and patient self-report of study data play a major role, but a critical review of available literature reveals that this assumption lacks support by published data [25]. Improvement in efficiency with eCRF may furthermore be affected by multiple factors that have not been studied so far such as length of CRFs, i.e. number of items in the CRF, complexity of items in CRFs and patients’ (e.g. age-related) ability to use eCRF. In summary, there are no studies that quantitatively assessed the time efficiency of pCRF and eCRF in a head-to-head comparison including the perspective of both involved parties, i.e. study personnel and patients, over multiple instruments in a CT [26]. Availability of such quantitative evidence is particularly important to estimate costs in planning of clinical trials and to support the implementation of an EDC system, which is associated with substantial costs [8, 27].

In this study, we evaluated how time efficiency and data quality are affected when source data is directly entered through eCRF into an EDC as compared to data capture with traditional paper pCRF including subsequent transcription to an eCRF. Secondary outcome parameter furthermore included predictors of efficient eCRF use.

## Methods

This study was conducted at a clinical research unit (CRU) of the Berlin Institute of Health, Berlin, Germany. It was approved by the local ethics committee review board and complied with local data protection regulations. The clinical trial was registered at ClinicalTrials.gov (NCT02649907). Particular attention was devoted to adherence to the *Helsinki declaration* [28].

### Participants

All patients and study personnel involved in a clinical weight loss trial conducted at our facility were eligible to be included in this study. There were no further inclusion or exclusion criteria. Study nurses gave verbal informed consent and patients provided written informed consent.

### Study design

This study was conducted in a randomized controlled parallel group design on top of a clinical weight loss trial at our facility. In order not to interfere with standard procedures, we collected data during routine visits of the main study. Patients and study nurses were randomly assigned to one of two data entry methods. A randomization list was created by one of the investigator (RF) using MATLAB R2008b (The MathWorks, Natick, MA, USA) and used to allocate participants to either the eCRF or pCRF group. This list contained six blocks of ten pCRF and ten eCRF slots that were shuffled using a uniformly distributed randomization function. With every visit, study nurses looked up the next entry in the randomization list, crossed out the entry and assigned the participant the respective entry method, i.e. tablet PC or paper and pencil. No stratification was used. Previous and subsequent list entries were visible for authorized study personnel but the designated order was strictly followed. Study nurses were not allocated one particular intervention, i.e. data entry method, but changed methods between visits. Participants, i.e. study nurses and participants, then filled all CRFs scheduled for the respective visit through the assigned method. The aim was to take 15 time records for all possible combinations of data entry methods and unique CRF types (study nurse CRF, patient short CRF, patient intermediate CRF, patient long CRF; see *materials* section for details), which resulted in a total number of 120 records, or 60 records for each data entry method, and a 3:1 participant to study nurse ratio. This sample size was chosen a priori since it is in line with previous studies in the field (e.g. 110 records by Salaffi et al., 75 records by Walther et al. [29, 30]) and resulted in a simple distribution of 15 records per groups times 2 data entry methods. A researcher took record of the data acquisition process and took care that not more than the required number of CRFs was collected through each data entry method, i.e. if only one data entry method was left for a certain CRF type, participants were automatically assigned this method.

**Fig. 1:**
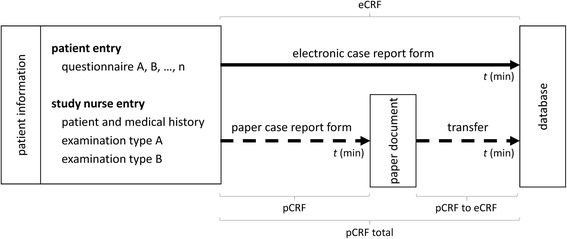
Summary of the study design. A nutritional assessment study served as use case to test our hypotheses regarding data collection methods in clinical trials. In this study, patient information was collected in two ways. Patients either entered information themselves into standardized instruments such as questionnaires or information about the patient was obtained by a study nurse and then entered. Entering patient information could be done in two ways that included either direct access to the digital database through an electronic case report form (eCRF) or indirect access by filling a paper based case report form (pCRF) that was subsequently transferred to the database (pCRF to eCRF). Precise records were taken for times required for data entry by either CRF type, subject type performing data entry and instrument type being used

The pCRF content was subsequently manually transcribed to the electronic database by a study nurse (pCRF to eCRF). In addition to data entry times and in order to test our hypotheses, each participant’s CRF entry method and age were documented (a summary of the study design is given in Fig. 1).

### Material

Eligibility criteria for CRFs from the main study to be included in this study were: fixed number of items irrespective of visit context (e.g. participant, intervention), comparable length of field content irrespective of visit context and possibility of continuous data entry without breaks. Ten CRFs fulfilled these criteria and were, in detail (item quantity in brackets): patient and medical history (292 items), examination at first visit (59 items), examination at second visit (18 items), visual analogue scale for subjective freezing (slider between 0 and 100 indicating subjective sensation of freezing, 3 items including metadata), nutrition questionnaire (28 items), international physical activity questionnaire (IPAQ, 38 items) [31], health survey (SF-36, 40 items including metadata) [32], chronotype questionnaire (43 items) [33], nutritional habits (52 items), test meal questionnaire (67 items). The first three CRFs were exclusively filled out by the study nurses while the remainder were exclusively answered by patients. Patient CRFs were subdivided into short (< 40 items), intermediate (40–60 items) and long (> 60 items) types and balanced between data entry methods to analyze the impact of CRF length on CRF entry efficiency (see *Analysis* in this section). Balancing was achieved through a researcher who took record of the data acquisition process and took care that the distribution of CRFs between data entry methods is equal, i.e. if a CRF was filled through one data entry method it had to be filled by a participant through the other method before the same method could be used again.

Participants used either tablet computers (iPad mini 4® or iPad Air 2®, Apple Inc., Cupertino, California, USA) to access eCRFs through the web interface of a research electronic data capture instance (REDCap, Vanderbilt University, Nashville, TN, USA; [34]) or used classic pCRFs. The transcription of pCRF data to eCRFs was performed by a study nurse through a REDCap instance running on a desktop computer. REDCap functionality included a module to perform a quality control verification on all fields in a project. Predefined (missing values, incorrect data type or values out of range, outliers for numerical fields, multiple choice fields containing invalid values) as well as user-defined rules were implemented to ensure data integrity. Study nurses finally checked each eCRF for data correctness and completeness before marking it as completed. Tablet functions were limited for patient use in such a way that patients were unable to switch between applications, use cameras or cloud services or to access data sets other than their own current instance. For data protection matters, no patient data were saved on tablets at any time; data were instead saved to a back-end that was accessible through the web interface. Data entry times were measured by using digital chronometers and results were written to standardized documentation sheets.

### Analysis

Participant characteristics were compared with an unpaired two-tailed t-test (age) or Pearson’s chi-squared test (male to female ratio). The primary hypothesis of this study was that data collection through eCRFs is faster than collecting data through pCRFs including subsequent manual data transcription to an electronic database and was tested by a two-tailed t-test for independent samples. We subsequently used t-tests to investigate how sub-procedures, i.e. pCRF data entry and pCRF data transcription, affected the total pCRF procedure and how these related to the direct eCRF entry method.

Secondary hypotheses were that data entry times would be less favorable for eCRFs in older patients and with fewer items due to relatively longer times required to load and render an eCRF. We tested these hypotheses in a step-wise approach. First, we set-up an analysis of covariance (ANCOVA) that allows for a regression analysis of continuous parameters. The ANCOVA included “time” as the dependent variable, the two-level group factors “CRF entry method” (eCRF, pCRF total) and “participant type” (study nurse, patient) as predictors and “item quantity” (continuous item numbers of each CRF) as a covariate. “Item quantity” was nested within “participant type” since each CRF was filled out by either study nurses or participants and never both. Post-hoc tests were performed by Fisher’s Least Significant Difference test. The influence of patients’ age on eCRF entry performance, i.e. time required to fill out a questionnaire, was tested on the patient data subset by regression analysis that included “time” as dependent variable and “patient age” and “item quantity” as predictors. Group data is reported as mean with its standard deviation with a precision of two decimal places throughout. *P*-values are rounded to three decimal places and values lower than .001 are not reported exact but as < .001.

Given that the above estimates of time savings result from a composite measure that includes both study nurses’ and patients’ CRF entry times, we conducted a second evaluation dedicated to estimate additional time saving effects of eCRFs resulting from patient self-report. To be more precise, time savings due to patient self-report of study data is not defined by a difference in pCRF and eCRF data entry but rather by the subsequent pCRF to eCRF transcription times of self-reported CRFs. The total time consumption divided by the total number of transcribed items reflects the time that study personnel saved per item. The time per item multiplied by the average quantity of CRF items containing patient reported data (constant) gives an estimate of how much time per CRF can be saved if source data was self-reported by patients.

To test data quality, we quantified the number of entry errors for each “CRF entry method” and compared absolute differences by descriptive statistics. Data entry or transcription were considered erroneous if the field type did not match the information provided (e.g. wrong date format), there were semantic or logic errors, or if fields were omitted.

All analyses were run using SPSS® Statistics (Version 23, IBM Corporation, Armonk, USA).

## Results

**Fig. 2:**
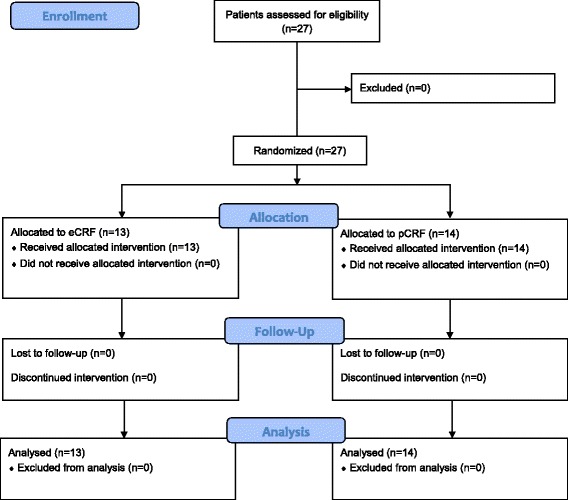
CONSORT 2010 Flow Diagram. This diagram illustrates that all patients that were assessed for eligibility to participate in this study also agreed to participate. Non of the included patients withdrew consent or decided not to use the randomized data entry method. Given the cross-sectional design without follow-up there was also no loss to follow-up. Study nurses were not included in this diagram since they were not allocated one particular intervention, i.e. data entry method, but changed methods between visits

Twenty-seven patients (57.1 ± 6.5 years old, 20 female, eCRF group, *n* = 13, pCRF group: *n* = 14) and two study nurses (36 and 49 years old, both female) were enrolled until data collection was completed. A CONSORT flow diagram of the patient enrolment procedure can be found in Fig. 2. Study nurses had two and three years of eCRF and pCRF user experience, respectively. None of the study nurses ever used mobile devices for eCRF data entry before this study. Information on patients’ user experience was unavailable since none but the original trial data was obtained. A total of 120 records including 8124 filled items were evaluated during the study period. A detailed description of data composition is given in Table 1. Patient groups did not differ with respect to age (*p* = .937) or male to female ratio (*p* = .686). A detailed description of participant characteristics is given in Table 2.

**Table 1:** Summary of data composition

	CRF entry method	
	eCRF	pCRF
Total number of records	60	60
Records per subject type		
Patient	45	45
Study nurse	15	15
Records per instrument		
Patient and medical history	6	6
Examination at first visit	3	3
Examination at second visit	6	6
VAS^a^ freezing	5	5
Nutrition questionnaire	5	5
IPAQ^b^	5	5
SF-36^c^	5	5
Chronotype questionnaire	5	5
Nutritional habits	5	5
Test meal questionnaire	15	15

Equal numbers of records were obtained for each CRF, subject and instrument type. Differences in record numbers between items were owed to the design of the use case study and controlled for in the statistical model

^a^visual analogue scale

^b^international physical activity questionnaire

^c^short form (36) health survey

**Table 2:** Demographic characteristics of participants

	CRF entry method	
	eCRF	pCRF
Patient characteristics		
Number of patients	13	14
Age (years)	57.54 ± 5.79	57.36 ± 5.91
Male/female	2/11	3/11
Study nurse characteristics		
Number of study nurses	2	2
Age (years)	36, 49	36, 49
Male/female	0/2	0/2

Participants were either patients enrolled in a clinical weight loss trial or study nurses capturing data of these patients. Until completion of data collection 13 patients were enrolled in the eCRF group and 14 patients in the pCRF group. Patient groups did not differ with respect to age (*p* = .937) or male to female ratio (*p* = .686)

### Predictors for case report entry times

**Fig. 3:**
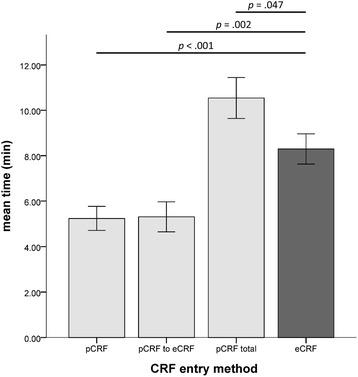
Plot of average time consumption for data entry procedures. Patients and study nurses could enter data to the database in two ways; they either entered data directly through eCRF or first to a pCRF whose content was subsequently transferred from pCRF to eCRF resulting in a pCRF total time. Results show that entering data through eCRF is significantly faster than the complete pCRF procedure (‑2.25 ± .99 min, *p* = .047). Errors bars represent standard deviation

The test for the primary hypothesis revealed that completing an eCRF was faster and took on average 8.29 ± 5.15 min as compared to an average of 10.54 ± 6.98 min necessary to complete the whole pCRF procedure (t_(118)_ = 5.87, *p* = .047). Decomposition of the pCRF procedure revealed that each single sub-step was significantly faster than completing an eCRF. Average times required were 5.23 ± 4.10 min (t_(118)_ = −3.59, *p* < .001) and 5.31 ± 5.11 min (t_(118)_ = −3.18, *p* = .002) for pCRF data entry and pCRF to eCRF data transcription, respectively (Fig. 3).

The full model ANCOVA revealed a significant main effect for CRF entry method on time consumption (F_(1,112)_ = 4.33, *p* = .040). There was no time difference, however, with regards to the participant type irrespective of CRF entry method (F_(1,112)_ = 2.36, *p* = .127) or the interaction between participant type and CRF entry method (F_(1,112)_ = .15, *p* = .699). These results do not provide evidence that the main effect was confounded by or confined to time saving effects in one participant type. Descriptive statistics of the primary outcome parameter grouped by participant types were as follows. The average time required for patients to complete an eCRF was 7.89 ± 4.17 min as compared to 10.28 ± 6.18 min for the pCRF procedure including transcription, which corresponds to a time saving effect of 23% by eCRF use. The average time required for study nurses to complete an eCRF was 9.49 ± 7.43 min as compared to 11.32 ± 9.2 min for the whole pCRF procedure, which corresponds to a time saving effect of 16% by eCRF use. The global test furthermore revealed a significant main effect of the CRF item quantity on data entry time (F_(2,112)_ = 34.80, *p* < .001) that was not specific to the CRF entry method (F_(2,112)_ = .49, *p* = .609).

In line with results from the ANCOVA, CRF item quantity was a significant predictor for data entry time in the regression model (Beta = .48, *p* = .001). Patient age, however, was not a significant predictor (Beta = .09, *p* = .534).

### Time saved for study personnel when patients use eCRF

Based on results we calculated estimates for the time that would be saved for study personnel engaged in data collection when patients used eCRFs instead of pCRFs. Results suggest that study personnel would save on average 6.88 ± 3.77 s per item or 5.16 ± 2.83 min per CRF assuming an average number of 45 items per CRF as was the case in our study.

### Effects of CRF entry method on data quality

The total number of data entry errors was only 3 in 120 records that were analyzed in this study. All three data entry errors occurred in the pCRF condition (3/60, 5%) and none in the eCRF condition (0/60, 0%). All entry errors were data that was omitted. There were no transcription errors.

## Discussion

This is the first study to demonstrate in direct comparison that the use of eCRFs in CTs including patient reported data capture facilitates time efficient data handling irrespective of CRF item quantity and patient age [26]. Data integrity was also higher in the eCRF condition (0 mistakes), yet this result is tentative as somewhat unexpectedly only 3 mistakes were made per 4000 total entries into the pCRFs.

### Effect of CRF entry method on data handling efficiency and comparison to previous studies

In our study, the use of eCRFs for patient-reported and nurse-reported measures lead to a 23% and 16% reduction of time consumption as compared to pCRF use, respectively. Decomposition of the pCRF procedure revealed that data entry per se was quick but transcribing the data into an EDC led to time savings with eCRFs. One could argue that the benefit of time savings due to unnecessity of data transcription can only be generated if data transcription was done manually and not performed through automated procedures, e.g. using publicly available scanning software including optical field and character recognition (OCR). However, both manual and automated transcription into an EDC are followed by additional time-consuming data validation [35]. While the necessity of a validation process does not explicitly imply that pCRFs require twice the amount of time as compared to eCRFs given equal data entry times, it does exemplify that direct data acquisition with eCRFs should be more time efficient in any scenario that involves transcription procedures.

There are few other studies that looked at the time saving effect of electronic case report forms. Schmidt et al. conducted an observational cross-over study on top of an open-label non-randomized Phase II study. They reported that replacing pCRFs by eCRFs decreased the time required for data entry by 59-69%, which substantially outperforms our findings [36]. Methodological aspects critically resolve this substantial difference to our results since Schmidt et al. included 48-69% of prepopulated data from the clinical information system (CIS) in their calculations. Data entry was furthermore exclusively performed by study personnel and times were not measured but estimated by participants, which poses a relevant bias that could not be controlled for, e.g. through blinding. Although exact time saving potential generated in the study by Schmidt et al. remains unclear given methodological shortcomings, the study highlights that prepopulating data from the CIS may further increase data handling efficiency in future studies.

In contrast to the results discussed above, a study by Walther et al., which was not conducted on top of a clinical trial and did not randomize the intervention, found no significant difference in time consumption between pCRFs and eCRFs (on various portable devices) [30]. Although total times for the pCRF condition were not reported as such, they found that filling out pCRFs took on average 17 min and data transfer about 15 min summing up to about 32 min of total pCRF time. In comparison, data entry times for eCRFs were 33 min for tablet computer use, 38 min for netbook use and 40 min for PDA (personal data assistant) use. Although no initial time saving effects were reported, they found that the time required to enter data to eCRFs decreased over the course of three weeks by roughly 30-50% depending on the type of method. Importantly, this study exclusively tracked times required by study personnel to enter telephone interview data. These results suggest that the complexity of data entry plays a role in the time efficiency of eCRFs. The authors argue that the lack of difference between methods might also be due to the fact that interview content text fields were overrepresented in their CRFs and that longer text passages are less difficult to enter with handwriting, i.e. into pCRFs. It would thus be plausible to assume that data-entry challenges play a predominant role in eCRF vs. pCRF efficiency. In our trial, we found no effect of item quantity, type or length of CRFs. This suggests that technical challenges were less predominant and is in line with the fact that our eCRFs were void of entrance fields for longer passages of text (see next paragraph for in depth discussion).

Thriemer et al. assessed the burden of community-acquired bloodstream infections in febrile patients utilizing technically simple tools running on PDAs and found a 50% reduction in time consumption per patient for eCRFs versus pCRFs (5 vs. 10 min, *n* = 2209 and 180, respectively) [37]. Unlike our study, this study only evaluated entry of routine clinical data performed by study personnel and was not conducted on top of a randomized clinical trial. Time saving effects were again substantial for eCRFs, even in the condition that benefitted least in our study, i.e. data entered by study personnel. One possible explanation is the method that was used to calculate time savings. In the study by Thriemer et al. only two of four CRF sections were converted to an efficient eCRF structure. Here, mainly checkboxes and radio buttons that benefit particularly from direct data entry were included. The remaining sections, which were difficult to implement in the eCRF design, were manually transcribed in both conditions. To calculate time savings an average of 1.4 min/page was assumed, i.e. transcription times were not measured but estimated. The reduction from 14 to 7 pages requiring transcription in the eCRF condition thus resulted in an estimated time saving potential of at least 50%. It is, however, unclear if transcription of the transcribed sections (2 section of PDA data entry) would indeed have doubled times. In contrast, we took precise time records of how eCRF use affects time efficiency when all pCRF based data entry is digitalized. This is more likely to be the daily routine of a clinical research unit and a real-world scenario.

### Effects of predictors other than CRF entry method on data entry time

To our knowledge, there are no other studies published in peer-reviewed journals that evaluated predictors such as patient age or CRF item quantity on data entry times in the context of two different methods, i.e. pCRF and eCRF. One study used an indirect method and assessed how total patient interview times were changed when pCRFs were replaced by eCRFs and did not find an age-dependent effect, yet it remains unclear how data entry as a subroutine of the patient interview was affected [29]. It has also not been established if patients performed similarly to experienced and trained study nurses. For example, one well-documented concern is that elderly patients are less comfortable and skilled using novel technology [38]. Regression analyses, however, clearly yielded that age was not a significant predictor of eCRF entry times in twenty-seven patients. Future studies should evaluate the subjective perception and preferred data entry method in order to get an idea if responses in a subset of patients might be affected by using novel technology.

It is furthermore self-evident that study personnel are better trained than patients and might thus particularly benefit from using eCRF, yet study nurses, and possibly the majority of patients, had no prior experience using mobile devices to enter study data. In support of this assumption, results do not suggest that participant type, i.e. study nurse or patient, is a relevant confounder. In contrast, post-hoc analyses revealed that patients tended to be more efficient than nurses using eCRF. The possibility of an underlying training effect could not be tested on the basis of this study’s data since the order in which patients answered CRFs was not recorded and CRF complexity may additionally confound sequence effects. Yet, it is possible that this effect is due to differences in the CRF content between subject types with patient questionnaires having a relatively larger amount of checkboxes and radio buttons requiring less time than text fields. This does not, however, rule out that training effects might play a more important role in longitudinal studies in which patients use eCRF multiple times and if study personnel become familiarized with the use mobile eCRF. Study nurses, like the patients, had no prior experience using mobile devices to enter source data.

The third secondary hypothesis we tested was that longer questionnaires might benefit more from digital data capture since time required to load and render eCRF is relatively shorter. Although number of items per CRF is self-evidently a significant predictor of data entry time, this effect was not specific for any particular CRF entry method.

### Estimates of time saved for study personnel

In an attempt to estimate how much time patient self-report saves study personnel, we extrapolated time savings due to data transcription redundancy. We estimated that this would save about 6 s per item or 5 min per questionnaire in this study. Since 7 patient questionnaires were included, time savings would sum up to about 35 min per patient and scheduled visit which implies substantial time and cost savings considering the complete study course. This secondary outcome data has yet to be interpreted carefully in the context of cost-efficiency since aspects such as providing the required infrastructure were not included. There is yet a retrospective meta-analysis of 27 studies available reporting substantial cost savings when eCRFs instead pCRFs are used [22].

### Data entry quality

Quantitative interpretations regarding data integrity are difficult to draw since the total number of data entry errors was generally low in this study. Yet, relative numbers are strikingly similar to published error rates of about 5% when using pCRFs and less than 1% when using eCRFs [30, 37]. Descriptive statistics are in line with published data indicating that eCRF data quality tends to be superior to pCRF data quality and facilitates collection of accurate and complete data [22]. The Food and Drug Administration acknowledge in their 2013 guidance on electronic source data in clinical investigations that prompts, flags, and data quality checks in eCRFs minimize errors and omissions during data entry, a view that is supported by our data [39]. They also argue that capturing source data electronically would eliminate unnecessary duplication of data, reduce transcription errors, facilitate data monitoring and promote real-time access for data review, assumptions that yet require validation in future studies.

### Limitations

Our analyses assume that using pCRFs inevitably renders manual data transfer to an electronic database necessary. The use of OCR scanners is an alternative method to import pCRF content. However, recent research has shown that these systems have an error rate of up to 20% and require substantial time effort to check data integrity [35]. A notable exception is the recognition rate of multiple choice radio buttons that was reported to be as high as 99.2%. This suggests that pCRFs thus might be a viable solution for simple questionnaires but not as a generic solution. Future studies might still decide to evaluate a head-to-head comparison of eCRFs and pCRFs in combination with OCR technology. Another limitation is that the full time saving potential of eCRFs was possibly underestimated since our system not yet included the possibility to directly draw CIS data. It is consistently shown that this option would allow prepopulating about 15% of clinical data directly and up to about 50% indirectly [36, 40]. It is furthermore important to note that the comparison of the eCRF performance of study nurses and patients is tentative since only two study nurses participated in the study and used both data entry methods unlike patients who were randomly assigned one method. It was impossible to include more than the two nurses in this study since these were the only two nurses with sufficient training to conduct the clinical trial on top of which we conducted our study. Future studies investigating the effect of training on eCRF data entry performance as a primary outcome parameter should include equal numbers of nurses and patients and randomize both groups.

## Conclusions

This study provides evidence that the use of mobile eCRF is associated with substantial time reductions for entering patient self-reported data and data collected by study personnel to an EDC system. This effect is not influenced by patient age, CRF item quantity or questionnaire variants. The use of eCRFs should be preferred over pCRFs in clinical research settings in terms of time efficiency, data quality and patient utilization.

## Abbreviations

CIS: Clinical information system
eCRF: Electronic case report form
EDC: Electronic data capture
OCR: Optical field or character recognition
pCRF: Paper case report form
PDA: Personal data assistant
